# Psychobiotics in Mental Health – Between Hope, Evidence, and the Need for Precision

**DOI:** 10.31083/AP45973

**Published:** 2026-04-09

**Authors:** Alberto Sá Filho, James Oluwagbamigbe Fajemiroye, Sergio Machado

**Affiliations:** ^1^Graduate Program in Pharmaceutical Sciences, Pharmacology and Therapeutics (PPGCFFT), Evangelical University of Goiás-UniEVANGÉLICA, Anápolis, GO 75083-515, Brazil; ^2^Institute of Biological Sciences (ICB), Universidade Federal de Goiás (UFG), Goiânia, GO 74690-900, Brazil; ^3^Laboratory of Physical Activity Neuroscience, Neurodiversity Institute, Queimados, RJ 26325-010, Brazil

Over the past two decades, the notion that the human gut microbiome could influence brain function has shifted from speculative curiosity to a central theme in psychiatric research. Within this context, psychobiotics, probiotics, prebiotics, and synbiotics capable of conferring mental health benefits [1] have attracted increasing attention as potential adjunctive strategies for the management of depression and anxiety. Unlike traditional pharmacological approaches, which act predominantly on monoaminergic neurotransmission, psychobiotics engage multiple biological systems simultaneously. They modulate inflammation, restore intestinal barrier integrity, influence tryptophan metabolism, regulate hypothalamic–pituitary–adrenal (HPA) axis activity, and alter the synthesis of neurotransmitters such as serotonin, dopamine, gamma aminobutyric acid (GABA), and norepinephrine [2]. Clinical trials have reported reductions in depressive and anxious symptoms, while preclinical models support anxiolytic-like effects across different experimental paradigms [3]. Equally important, psychobiotics are generally safe, well tolerated, and carry none of the adverse long-term effects often associated with conventional psychotropics.

Multiple pathways converge to support their potential role in mood regulation. Specific *Lactobacillus* and *Bifidobacterium* strains enhance serotonergic and GABAergic neurotransmission, bacterial metabolism of tryptophan counterbalances the kynurenine pathway to reduce neurotoxic metabolites, and modulation of the HPA axis attenuates cortisol responses to stress [4]. Anti-inflammatory actions are consistently reported, with psychobiotics promoting interleukin-10 (IL-10) and suppressing proinflammatory cytokines such as IL-6 and tumor necrosis factor-alpha (TNF-α) [5]. Beyond immunomodulation, short-chain fatty acids produced by gut microbes act epigenetically as histone deacetylase inhibitors, promoting neuroplasticity and stress resilience. Novel findings on bacterial extracellular vesicles suggest additional routes of communication between gut microbes and host brain function. Collectively, these insights reinforce the concept that psychobiotics can serve as a biological bridge between the gut and brain in the context of depression and anxiety.

However, enthusiasm must be tempered by caution. The current evidence is marked by heterogeneity, methodological fragility, and frequent contradictions. The publication of 33 systematic reviews and meta-analyses [4, 6] (just in the last 10 years) provides one of the most comprehensive assessments to date of psychobiotics in mental health. The findings are both promising and sobering. On one hand, meta-analyses often reveal statistically significant reductions in depressive or anxious symptoms [4, 5, 6], particularly when psychobiotics are administered as adjuncts in clinically diagnosed populations. On the other hand, the magnitude of the effect is modest, frequently context-dependent, and far less convincing in healthy individuals or when psychobiotics are used as monotherapy. Methodologies are also inconsistent. In the application of the A MeaSurement Tool to Assess systematic Reviews (AMSTAR)-2 instrument of assessments, only a small minority of reviews achieved high confidence, while most were rated as critically low, reflecting flaws such as absence of pre-registered protocols, inadequate risk of bias assessments, and failure to consider publication bias. Application of the GROOVE tool [7] revealed a corrected covered area of nearly 14%, highlighting substantial redundancy across reviews and emphasizing that much of the secondary literature reiterates the same primary trials rather than contributing to novel insights.

There continue to be significant limitations of the evidence base. Many early clinical trials were conducted on healthy volunteers, diluting the apparent efficacy in populations with clinically significant disorders. Standardization is notably absent: there is no consensus on which strains, combinations, or dosages should be prioritized. The vast majority of interventions are short term, lasting between four and twelve weeks, leaving questions unanswered about the durability of effects, relapse prevention, and long-term safety. Clinical outcomes are fragmented, with discrepancies between self-reported scales such as the Beck Depression Inventory and clinician-rated measures such as the Hamilton Depression Rating Scale, leading to inconsistent interpretations. Furthermore, clinical studies on psychobiotics rarely include populations with significant treatment gaps, such as older adults with comorbidities or treatment-resistant patients, and often overlook gender-related differences in response to treatments [4, 5, 6].

The way forward requires a deliberate shift toward what can be termed “precision psychobiotics”. This means targeting clinical populations most likely to respond, abandoning generalized approaches in favor of strain-specific recommendations, and recognizing that multi-strain formulations have consistently outperformed single-strain products. Identifying the optimal therapeutic window is essential, with current evidence indicating that 4- to 8-week interventions may be most effective [8]. Psychobiotics are best positioned as adjuncts to pharmacological and psychotherapeutic treatments rather than stand-alone alternatives. It also requires trials with longer follow-up to assess sustainability, biomarker-guided designs to stratify responders from non-responders, and rigorous methodology that avoids redundancy and strengthens reproducibility [4, 5, 6, 8].

Psychobiotics stand at a critical crossroads. Their biological efficacy is compelling [2], their safety profile reassuring, and their early clinical signals promising. Yet, the evidence remains insufficient for unconditional endorsement. The challenge is no longer to prove their potential, but to refine their application. Without precision, psychobiotics may be interpreted as an overestimated intervention. With rigor and careful targeting, they can become an integrative therapy in psychiatry, bridging microbiology, neuroscience, immunology, and clinical psychology.

Psychobiotics embody the relationship between hype and hope. The promise is real, but so are the limitations. Their future lies not in broad, unspecific claims, but in carefully delineated contexts where biological plausibility, methodological rigor, and clinical need converge. If that path is pursued, psychobiotics may indeed transition from experimental adjuncts to established tools of personalized psychiatry. Until then, despite the 33 systematic reviews and meta-analyses, they remain a field where evidence must be strengthened, precision embraced, and enthusiasm balanced with caution.
